# Signaling networks in immunometabolism

**DOI:** 10.1038/s41422-020-0301-1

**Published:** 2020-03-20

**Authors:** Jordy Saravia, Jana L. Raynor, Nicole M. Chapman, Seon Ah Lim, Hongbo Chi

**Affiliations:** Department of Immunology, St. Jude Children’s Research Hospital, Memphis, TN 38105 USA

**Keywords:** Innate immunity, Autoimmunity

## Abstract

Adaptive immunity is essential for pathogen and tumor eradication, but may also trigger uncontrolled or pathological inflammation. T cell receptor, co-stimulatory and cytokine signals coordinately dictate specific signaling networks that trigger the activation and functional programming of T cells. In addition, cellular metabolism promotes T cell responses and is dynamically regulated through the interplay of serine/threonine kinases, immunological cues and nutrient signaling networks. In this review, we summarize the upstream regulators and signaling effectors of key serine/threonine kinase-mediated signaling networks, including PI3K–AGC kinases, mTOR and LKB1–AMPK pathways that regulate metabolism, especially in T cells. We also provide our perspectives about the pending questions and clinical applicability of immunometabolic signaling. Understanding the regulators and effectors of immunometabolic signaling networks may uncover therapeutic targets to modulate metabolic programming and T cell responses in human disease.

## Introduction

The adaptive immune system is comprised of T and B cells and is critical for host responses to invading pathogens and tumors. Upon T cell receptor (TCR) recognition of the cognate antigen in the presence of co-stimulatory and cytokine signals, signaling networks are activated in naïve T cells to facilitate clonal expansion, effector cell differentiation and immune function. Following antigen eradication, most effector cells die via programmed cell death, but some cells persist long-term as memory cells and are primed for rapid recall response. Activation states and subpopulations of T cells account for marked cellular heterogeneity. In addition, T cell functional states have shared and distinct transcriptional programs, along with differences in protein expression, activity and interactions that are only beginning to be understood.^1^

In recent years, the importance of cellular metabolism in dictating T cell development and function has become increasingly realized. Quiescent cells, such as naïve or memory T cells, favor mitochondria-driven catabolic metabolism, including oxidative phosphorylation (OXPHOS).^2^ Upon activation, T cells undergo quiescence exit driven in part by the catalysis of nutrients, such as glucose and glutamine, which serves to generate sufficient macromolecular supply for cellular growth and support the increased energy demands associated with such growth. This metabolic reprogramming not only includes elevated aerobic glycolysis and glutaminolysis, but also a marked upregulation of mitochondrial biogenesis and function.^2^ Furthermore, these changes in metabolic programs must be properly regulated to maintain immune homeostasis and functional specificity of T cell subsets.^1,2^ How metabolic programs are modulated by upstream signaling networks and the mechanistic role of metabolic rewiring in T cells therefore remain critical to address.

In this review, we discuss cellular signaling pathways involved in immunometabolic regulation, including relevant molecules, upstream and downstream targets and their cell type-specific effects. Specifically, we focus our discussion on phosphoinositide 3 kinase (PI3K) — protein kinase A, G and C (AGC) kinases, mechanistic target of rapamycin (mTOR) and liver kinase B1–5′ AMP-activated protein kinase (LKB1–AMPK) signaling. We also comment on emerging research interests within these pathways and the potential areas for future investigation. Thus, this review provides necessary information on molecular signaling, contextualized for the immunometabolism field, especially in T cells, and highlights how this field may drive advancements in therapies for human disease.

## PI3K–AGC signaling

**Fig. 1:**
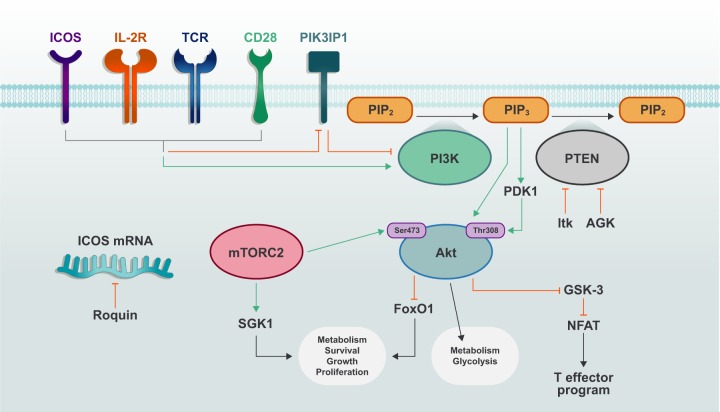
PI3K–AGC signaling in T cell activation and metabolic reprogramming. Activation of TCR, CD28 and IL-2R induces phosphorylation and activation of PI3K and also inactivation of PI3K-suppressing molecules, such as PTEN and PIK3IP1. PIP_2_ is converted to PIP_3_ via the activity of PI3K, and PIP_3_ facilitates plasma membrane recruitment and activation of downstream signaling molecules including PDK1 and Akt. mTORC2 further activates Akt and promotes increased metabolism and T cell effector function.

Phospholipids are key second messengers that influence downstream immunometabolic pathways and are thus highly regulated with respect to turnover. One central mediator of phospholipid turnover is PI3K, which converts phosphatidylinositol-(4,5)-bisphosphate (PIP_2_) into phosphatidylinositol-(3,4,5)-trisphosphate (PIP_3_). Generation of PIP_3_ allows for plasma membrane recruitment and functional modulation of proteins containing pleckstrin homology (PH) domains. Thus, PI3K activity can generate a subcellular hub of signaling by recruiting numerous PH domain-containing effector proteins into close proximity. PI3K activity induces diverse signaling pathways involved in regulating cellular function, including Akt (also called protein kinase B), phosphoinositide-dependent protein kinase 1 (PDK1), mTOR complex 1 (mTORC1) and mTORC2 (mTORC1 and mTORC2 discussed in a separate section below). In this section, we will describe the PI3K-dependent signaling networks, especially those related to activation of the AGC kinases, in T cells and how they influence cellular metabolism (Fig. 1).

### PI3K

Class I PI3Ks are heterodimeric proteins consisting of a catalytic p110 subunit (p110α, p110β, p110δ, or p110γ) and one of five regulatory subunit isoforms (p50α, p55α, p55γ, p85α, or p85β). In the absence of stimulation, the kinase domain of the catalytic subunit is suppressed by the regulatory subunit.^3^ The concentrations of PI3K substrates are closely linked to cellular activation state, which, in T cells, is dictated by TCR engagement, CD28 family-mediated co-stimulation and cytokine signaling, such as downstream of IL-2 receptor (IL-2R).^2^ Following upstream activation, the Src-homology-2 (SH2) domain of PI3K can bind specific phosphorylated YXXM motifs on upstream receptors or adapter proteins, causing the release of inhibitory connections of the regulatory subunit and the translocation of the catalytic subunit to the substrate-rich plasma membrane.^4,5^ T cell activation is associated with a shift in signaling and transcriptional programs, which enhances anabolic metabolism, fueled by catabolic processes (e.g., glycolysis and glutaminolysis), to meet the increased demand for cellular nutrients and energy. This exit from quiescence is crucial for the development of effector T cell populations.^1^ Indeed, PI3K activation is a seminal event in this process, as its downstream signaling cascades result in elevated aerobic glycolysis and mitochondrial biogenesis,^2,6^ as discussed more below.

PI3K signaling is negatively regulated by phosphatase activity. Specifically, PIP_3_ is converted to PIP_2_ or PI-(3,4)-P_2_ by phosphatase and tensin homolog (PTEN) and SH2 domain-containing inositol 5′-phosphatase (SHIP), respectively. SHIP plays an essential role in promoting Th1 cell responses,^7^ whereas PTEN deficiency leads to hyperactivation of T cells, especially under suboptimal stimulation.^8^ PTEN is repressed upon strong TCR stimulation or CD28 co-stimulation, in part, due to the Tec family kinase IL-2-inducible T cell kinase (Itk),^8–11^ and is also negatively regulated by acylglycerol kinase (AGK)-mediated phosphorylation during CD8^+^ T cell activation.^12^ Mice with T cell-specific PTEN deficiency develop spontaneous autoimmunity associated with hyperactivation of T cells as noted above, as well as lymphoma.^13^ Regulatory T (Treg) cell-specific PTEN deficiency also results in autoimmunity due to destabilized Foxp3 expression (also called instability) and diminished suppressive function of Treg cells.^14,15^ PTEN deficiency in Treg cells is associated with altered glycolytic and mitochondrial metabolism, as well as changes in DNA methylation at conserved noncoding sequences that are essential for Treg cell stability.^14,15^ T cells lacking Itk, and thus the ability to suppress PTEN function upon T cell activation, display increased Treg cell differentiation in vitro and in vivo.^9^ Thus, negative regulation of PI3K signaling is associated with proper metabolic regulation for control of immune homeostasis and suppression of tumorigenesis.

The p110 catalytic subunit of PI3K can be directly inhibited by the transmembrane protein PI3K-interacting protein 1 (PIK3IP1). T cells downregulate PIK3IP1 expression following activation, suggesting its importance in restraining PI3K signaling until proper activation signals are received. Indeed, T cells lacking PIK3IP1 display increased activation and IL-2-driven Th1 cell proliferation.^16,17^ PI3K signaling is also regulated at the mRNA level by Roquin proteins, which limit translation of target mRNAs encoding co-stimulatory receptors, such as ICOS.^18^ Mice with Roquin deficiency develop lupus-like autoimmunity with uncontrolled T follicular helper (Tfh) cell responses.^18^ Interestingly, Treg cell-specific loss of Roquin causes increased T follicular regulatory cells, which are capable of limiting germinal center (GC) responses but not colitis.^19^ Thus, PIK3IP1 and Roquin restrain T cell responses under selective contexts, unlike the more generic suppression mediated by PTEN.

The role of the class III PI3K (catalytic subunit vacuolar protein sorting 34 (Vps34)) in immunometabolic signaling is less understood, although it is important for autophagy (discussed more below) and vesicle trafficking in certain cell types.^3^ Interestingly, T cell development does not require Vps34, but survival of naïve T cells is severely impaired in its absence, likely due to improper recycling of the pro-survival cytokine receptor for IL-7 (IL-7R).^20,21^ Also, Vps34-deficient T cells display enhanced mitochondrial accumulation due to reduced mitochondrial clearance,^22^ which may also contribute to reduced survival. Together, these results demonstrate that PI3K signaling supports metabolic programming to orchestrate the homeostasis and function of T cells.

### AGC kinases

Among the most notable PH domain-containing proteins are members of the AGC kinases, which include PDK1, Akt, ribosomal S6 kinase (RSK, also called p90) and serum/glucocorticoid-regulated kinase 1 (SGK1).^3^ PDK1 is a serine/threonine kinase with important roles in activating other AGC kinases, including Akt and SGK1 (discussed below). In the absence of PDK1, T cell activation, but not survival, is reduced, in part due to defective NF-κB activation.^23^ TCR and CD28 activation promotes PDK1 recruitment to the plasma membrane and induces its phosphorylation, which is antagonized by PIP_2_ or inhibition of PI3K activity. Protein kinase C-θ (PKC-θ)-dependent phosphorylation of PDK1 at Thr513, rather than the auto-phosphorylation of the kinase domain residue (Ser241), is essential for driving T cell activation.^23^ In activated CD8^+^ T cells, PDK1 is involved in metabolic reprogramming that sustains glucose uptake and glycolysis downstream of IL-2 stimulation; this process requires the mTOR–HIF-1α (hypoxia-inducible factor-1α) axis, but not PI3K or Akt activity,^24,25^ although Akt is essential for programming glucose uptake in other T cell subsets as discussed below. In addition, Treg cells deficient in the autophagy protein Atg7 have increased PI3K–PDK1 signaling, which is associated with heightened mTORC1 activity, glycolysis and the development of an autoimmune disorder.^26^ On the other hand, deficiency for PDK1 in T cells leads to chronic intestinal inflammation, due to an imbalance of CD8^+^ γδ T cell accumulation; colitis in these mice is attributed to reduced Treg cell function in the absence of PDK1.^27^ Thus, the proper regulation of PDK1 activity is essential for enforcing appropriate T cell activation, as well as Treg cell function for the control of inflammation.

Akt is the best-studied AGC kinase in immune cells, whose activation is induced upon its recruitment to the plasma membrane. Maximal Akt activation is achieved following phosphorylation by mTORC2 at Ser473, which allows Akt binding to the substrate dock, termed the ‘PIF pocket’, of PDK1, which promotes phosphorylation of Akt at Thr308.^28^ mTORC2–Akt signaling coordinates T cell activation, differentiation and trafficking.^24,29–31^ In addition, Akt activates processes associated with cellular metabolism, such as by increasing glycolysis during the later stages of TCR and co-stimulatory receptor activation.^32,33^ This function may be mediated by increased phosphorylation of glucose transporter 1 (GLUT1) by Akt, which promotes its transport to the plasma membrane.^34,35^ One notable observation is that Akt is essential for programming initial activation of glycolysis in memory, but not naïve, T cells,^32,36^ but how Akt signaling differentially regulates the timing of glucose metabolism among T cell subsets or during different states of activation is unknown.

One important role for Akt in T cells is to regulate the activity of forkhead box O (FoxO)^37^ transcription factors by phosphorylation, which leads to their exclusion from the nucleus and termination of target gene transcription. FoxO proteins are more transcriptionally active in quiescent cell populations such as naïve T cells, where they promote expression of the homeostatic cytokine receptor IL-7R and also cell trafficking molecules (e.g., CD62L, CCR7 and S1PR1) via KLF2.^37–39^ Mice with T cell-specific FoxO1 deficiency develop mild autoimmunity, in contrast to the severe, lethal autoimmunity in mice with Treg cell-specific FoxO1 deficiency.^40^ Interestingly, more recent evidence suggests that proper downregulation of FoxO1 activity is crucial for T cell homeostasis, as mice harboring a constitutively active FoxO1 mutant in T cells also develop severe autoimmunity.^41^ Mechanistically, FoxO1 downregulation in activated T cells is important for the coordination of cellular growth and proliferation by allowing sustainable mTORC1 signaling and anabolic metabolism.^41^ In Treg cells, ‘graded’ increases in FoxO1 activity has context-specific functions; constitutive FoxO1 activity impairs Treg cell-dependent suppression of lymphoproliferative disease driven by CD8^+^ T cells, whereas partial enhancement of FoxO1 activity reduces Treg cell accumulation in non-lymphoid tissues, including tumors, leading to increased anti-tumor T cell activity.^42^ Thus, the Akt–FoxO1 axis dynamically regulates T cell responses.

Another serine/threonine kinase involved in Akt signaling is glycogen synthase kinase 3 (GSK-3), which is constitutively active in resting cells, but is inactivated via phosphorylation by Akt.^43^ Active GSK-3 plays an important functional role in T and B cells to promote their survival and to restrict activation, by limiting nuclear factor of activated T cells (NFAT) activity.^44^ However, GSK-3 function is not limited to naïve or quiescent cells, as it is also a scaling factor for anabolic metabolism in cells with ongoing or elevated metabolic profiles, such as GC B cells.^45^ The context-specific roles of GSK-3 will therefore be interesting to investigate.

The precise roles of AGC kinases such as RSK, as well as their substrates, in T cell metabolic programming is of continued interest. The role of SGK1 is discussed in a later section.

### Emerging perspectives

PI3K–AGC signaling remains an important area of immunology research, as we continue to illuminate its potent influences on immune cell differentiation and magnitude of effector/memory T cell responses. This information has been a central focus of translational immunology research. Specifically, how is T cell-intrinsic PI3K signaling affected within the tumor microenvironment, and can modulation of certain PI3K factors improve the efficacy of treatments, such as adoptive cell therapy?

The transcription factor TCF-1 (encoded by *Tcf7*) has garnered considerable attention recently due to its role in preserving a stem cell-like state (termed ‘stemness’) in T cell populations during chronic inflammation or in the tumor microenvironment.^46–50^ In fact, asymmetric PI3K distribution during cell division can impart memory- or effector-like functional identity in daughter cells through differential regulation of TCF-1.^51^ This PI3K–TCF-1 axis plays a major role in tumor-infiltrating lymphocyte function; elevated extracellular potassium levels within the tumor microenvironment can stifle PI3K signaling, resulting in reduced T cell effector function needed for tumor clearance.^46,47^ Interestingly, this effect is also associated with elevated TCF-1 expression and stem cell-like phenotypes, including self-renewal and persistence.^46^

Chimeric antigen receptor T (CAR-T) cells are a promising anti-cancer therapeutic strategy, yet issues with relapse and non-responders to CAR-T cell therapy highlight that additional work is needed to optimize this treatment. Inhibition of PI3K–Akt signaling during CAR-T cell generation ex vivo results in a more central memory phenotype, and these types of CAR-T cells display superior anti-tumor efficacy.^52,53^ Furthermore, targeting PI3K for anti-tumor therapy is not reserved for CD8^+^ T cells, as specific inhibition of the PI3K isoform p110δ affects Treg cells more than conventional CD4^+^ and CD8^+^ T cells.^54^ Finally, myeloid cell-specific PI3K modulation may also become an important strategy in combination with checkpoint therapy, as targeting p110γ limits the suppressive activity of tumor infiltrating myeloid cells,^55,56^ which may indirectly impact T cell function in the tumor microenvironment.

The downstream target repertoire of PI3K-dependent kinases is a topic of renewed interest, especially for cell type or context specificity. For example, PDK1 and Akt dictate differences in transcriptional programs in activated CD8^+^ T cells, with PDK1 being essential for IL-2-induced glucose metabolism and proliferation and Akt being critical for cellular trafficking and IFN-γ production.^24^ Also, TCR signal strength differentially regulates Akt activity in CD4^+^ T cells, and, more importantly, the induction of Akt-dependent targets, which is associated with their differentiation into pro-inflammatory effector cells or Treg cells in vitro.^57^ In GC B cells, Akt has altered activity compared to non-GC B cells due to phosphorylation site dominance and a preferential induction of negative regulators of B cell receptor (BCR) signaling^58^; this study helps explain how GC B cells have repressed BCR signaling, despite utilizing the same signaling molecules as non-GC B cells. The concept of “same molecules, new pathways” is an exciting idea for the field of immunometabolic signaling and may help shed light on existing conflicts in the literature. Moreover, it may help to define how similar receptor-mediated signaling pathways can impart different functional programs in distinct immune cell subsets, which may also be mediated by alterations in metabolite composition driven by these pathways.

In addition to PI3K, other phospholipid-modifying enzymes modulate T cell function. For example, phospholipase C-γ1 (PLC-γ1), which is activated upon TCR engagement, enzymatically cleaves PIP_2_ into diacylglycerol (DAG) and inositol-(1,4,5)-trisphosphate (IP_3_). IP_3_ binds to receptors on the endoplasmic reticulum (ER), causing Ca^2+^ efflux from the ER and subsequent opening of Ca^2+^ influx channels on the plasma membrane. Elevated cytosolic Ca^2+^ concentrations directly promote activation of the phosphatase calcineurin, which dephosphorylates NFAT, allowing for its nuclear translocation. This signaling cascade is an important regulator of T cell proliferation by promoting glycolysis and OXPHOS.^59^ In turn, the induction of glycolysis can feedforward enhance Ca^2+^–NFAT signaling to further support T cell function.^60^ The turnover of DAG is also an important regulator of T cell responses and is mediated by the DAG kinases (DGK), which converts DAG to phosphatidic acid; deficiency for DGK is associated with activation of mTOR activity in T cells.^61,62^ Thus, how other lipid-related signaling networks control metabolic reprogramming will be an important area to address in future studies.

## mTOR signaling

mTOR is a serine/threonine protein kinase present in two signaling complexes, mTORC1 and mTORC2, which are composed of distinct protein binding partners.^63^ mTORC1 is defined by the mTOR kinase, the adapter protein regulatory-associated protein of mTORC1 (Raptor) and mammalian lethal with SEC13 protein 8 (MLST8; also called GβL), as well as the inhibitory subunits proline-rich Akt substrate-1 (PRAS1) and its target DEP domain-interacting protein (DEPTOR). In contrast, mTORC2 is composed of mTOR, rapamycin-insensitive companion of mTOR (Rictor) and DEPTOR, as well as the regulatory proteins, mammalian stress-activated protein kinase-interacting protein 1 (mSIN1) and protein observed with Rictor-1 or -2 (Protor1/2).^63^ Due to its ability to bind to the FKBP12–rapamycin complex, mTORC1 activity is more sensitive to inhibition by rapamycin than mTORC2, although both complexes are inhibited upon chronic or high-dose rapamycin treatment.^64^

mTORC1 signaling is essential for T cell development in the thymus, homeostasis in the periphery and differentiation into effector CD4^+^ Th1, Th2 and Th17 cells, as well as cytotoxic CD8^+^ T cells.^2,29–31,65–69^ By contrast, mTORC2 activity is more selectively required for Th1 and Th2 cell differentiation,^29,30^ but also regulates migration of Tfh and Treg cells.^70,71^ mTOR signaling plays an additional role in the antagonism of conventional T cell responses, by regulating the activation, lineage stability and suppressive function of Treg cells in vivo.^14,15,26,72–75^ Finally, mTOR signaling antagonizes the differentiation of long-lived memory CD8^+^ T cells in lymphoid tissues but promotes their development in non-lymphoid tissues.^76–80^ Thus, mTOR signaling is a central regulator of T cell responses.

**Fig. 2:**
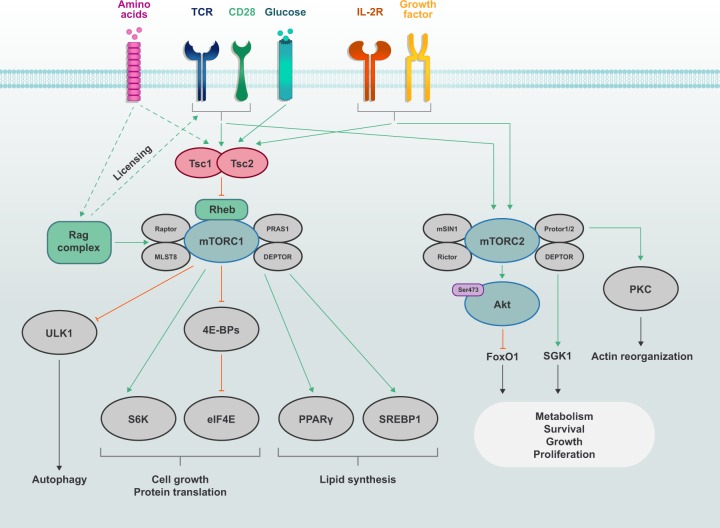
mTOR signaling in T cells. The discrete mTOR complexes, mTORC1 (consists of mTOR, Raptor, PRAS1, DEPTOR and MLST8) and mTORC2 (consists of mTOR, Rictor, Protor1/2, mSIN1 and DEPTOR), are activated by immunological receptors (TCR, CD28 and IL-2R) and growth factors. mTORC1 activation is also sensitive to nutrients such as amino acids. Amino acids promote mTORC1 activation through the Rag complex, which also plays a permissive ‘licensing’ role to allow for TCR and CD28 co-stimulatory signals to induce mTORC1 activation. The Tsc complex, whose activity is suppressed by immune and growth factor signals, inhibits the activation of the small G protein Rheb, which promotes mTORC1 activation. mTORC1 induces cell growth and protein translation through S6K and eIF4E, as well as lipid synthesis through PPARγ and SREBP1. mTORC1 inhibits autophagy through ULK1 under nutrient-replete conditions. In contrast, mTORC2 is activated by growth factors and it mainly regulates survival and actin reorganization via Akt, SGK1 and PKC. mTORC2 also plays critical roles in context- or subset-specific metabolic reprogramming for cell growth, proliferation and survival.

mTOR signaling is involved in many cellular processes, with a notable role in promoting protein synthesis through its phosphorylation of ribosomal protein S6 kinase (S6K; upstream kinase for ribosomal protein S6 (S6)) and eukaryotic translation initiation factor 4E (eIF4E) binding proteins (4E-BPs). On the other hand, mTORC2 induces Akt Ser473 phosphorylation, as well as the phosphorylation of PKC and other AGC kinases. Through these actions, mTORC2 promotes cell proliferation and survival^63^ and also has reported roles in cellular migration.^63,70,71^ Among its functions, the role of mTOR as a central regulator of cellular metabolism is of high interest in T cells. In this section, we will highlight the mechanisms and functions of mTOR signaling in reprogramming cellular metabolism in T cells (Fig. 2).

### Metabolic and immunological inputs for mTORC1

Lysosomes are a key signaling hub for driving mTORC1 activity. Several protein activators of mTORC1 are localized to the lysosome, including the small G proteins Ras homolog enriched in brain (Rheb) and Rag (obligate heterodimers comprised of RagA or RagB paired with RagC or RagD), which associate with the lysosomal membrane via the LAMTOR complex.^81–83^ Growth factors or receptor tyrosine kinases induce mTORC1 activity by inhibiting the GTPase activating protein (GAP) function of the tuberous sclerosis complex (Tsc; comprised of Tsc1, Tsc2 and Tbc1d7); the inhibitory phosphorylation of Tsc2 is usually mediated by Akt, but may be driven by RSK or extracellular signal-related kinase-1 or -2 (Erk1/2).^84–87^ Consequently, Rheb retains its GTP-bound state, which, when transiently recruited to the lysosomal membrane, allows Rheb to promote mTORC1 activation.^88,89^ TCR and CD28 co-stimulatory signals act as a major network that allows for inhibitory phosphorylation of Tsc2, whose deletion leads to spontaneous T cell activation and altered T cell homeostasis.^68^ However, other pathways, including insulin or Wnt signaling, may also promote mTORC1 signaling for T cell activation, differentiation or function.^63,90,91^ The precise mechanisms that dictate mTORC2 activity are unclear, although its activation is mediated by PI3K-induced PIP_3_ binding to mSIN1,^92,93^ thus allowing for increased mTORC2 activation under physiological conditions where AGC kinase function is likely to be essential.

Amino acids are a major nutrient signal that induces mTORC1 activation. Selective amino acids are stored in lysosomes, where under nutrient-sufficient conditions, they efflux into the cytoplasm via lysosomal v-ATPase, whose activity is regulated by SLC38A9 when SLC38A9 binds to the amino acid arginine.^94,95^ Upon amino acid stimulation, the Rag proteins convert to their active state (RagA- or RagB-GTP with RagC- or RagD-GDP), thus allowing binding to Raptor and recruitment of mTORC1 to the lysosome where it is engaged by Rheb.^82,96^ Amino acids are ‘sensed’ by upstream complexes that activate the Rag complex, including sestrin (binds leucine) and CASTOR1 (binds arginine), which trigger inactivation of the GATOR2 complex that suppresses Rag function.^97–102^ Leucyl-tRNA synthetase (LRS) and folliculin (Flcn), which binds folliculin-interacting protein (Fnip; isoforms Fnip1 and Fnip2), sensing systems have also been reported to promote Rag complex activation, by serving as GAPs for RagC or RagD^103,104^; notably, LRS can also activate Vps34 to stimulate the phospholipase D-dependent activation of mTORC1^105^ and may also promote the GTP binding of RagA or RagB via its GTPase GATOR1, although this effect has thus far only been identified in yeast.^106^ Glutamine can also indirectly induce Rag complex activity upon its conversion into α-ketoglutarate (α-KG) during glutaminolysis, which may be driven by leucine-dependent activation of glutaminase (GLS).^107^ Other nutrients, such as glucose and cholesterol, can prime mTORC1 signaling in a Rag complex-dependent manner, in part, through communication with SLC38A9 or the Neiman-Pick C1 complex,^96,108^ which requires further investigation in T cells.

Amino acids are also reported to regulate mTORC1 activation independently of the active Rag complex. For example, glutamine activates mTORC1 via ADP-ribosylation factor 1.^109^ Acetyl co-enzyme A (acetyl-CoA) derived from leucine can also modify Raptor via protein acetylation, such that its expression is stabilized to allow for mTORC1 activation.^110^ The ability of glutamine to promote mTORC1 activation upon TCR stimulation requires the CBM (CARMA1–Bcl10–MALT) complex, although the mechanisms are unclear as this is independent of canonical NF-κB signaling.^111^ In addition, the Tsc complex is displaced from the lysosome in the presence of amino acids, especially arginine, in activated Treg cells (and possibly conventional T cells), thus allowing for activation of mTORC1 signaling.^112^ Therefore, there appears to be coordinated communication between amino acid sensing and regulation of the GAPs that inhibit mTORC1 activation.

Recent work has established baseline differences in the expression of nutrient transporters between naïve CD4^+^ and CD8^+^ T cells.^113^ Upon stimulation by immunological signals, expression of nutrient transporter proteins increases to facilitate nutrient influx (with the degree of upregulation displaying differences in CD4^+^ and CD8^+^ T cell subsets^113^), including upregulation of ASCT2 (transporter for glutamine) and the LAT1–CD98 complex (transporter for leucine or other neutral amino acids).^111,114^ The increased expression of these transporters is essential for not only driving metabolic reprogramming as discussed below, but also inducing or maintaining mTORC1 signaling. Indeed, the induction of leucine- and glutamine-driven mTORC1 activation is impaired in LAT1- and ASCT2-deficient T cells, respectively,^111,114^ and mTORC1 activation is also reduced in CD98-deficient Treg cells, probably due to reduced isoleucine influx.^115^ Intriguingly, amino acids are also critical for promoting TCR- and CD28-dependent mTORC1 signaling in Treg cells,^112,116^ suggesting that amino acids and, possibly other nutrients, not only promote mTORC1 activation, but are also a prerequisite for immunological receptors to induce (a process we have termed ‘licensing’^112^) and sustain activity of mTORC1. Whether other nutrients also prime immune receptor signaling pathways at the level of mTORC1 or other signaling pathways remains unexplored.

### Glycolysis and mitochondria-associated metabolism

Cellular metabolism is a critical mediator of T cell responses.^2^ mTORC1 signaling promotes metabolic reprogramming toward increased aerobic glycolysis, glutaminolysis and remodeling of mitochondrial metabolism.^2^ Upon T cell activation, mTORC1 upregulates expression of the transcription factor c-Myc, whose activity is essential for promoting expression of genes involved in glucose and glutamine metabolism, including hexokinase 2 (rate-limiting enzyme for glycolysis) and GLS; the induction of these c-Myc-dependent programs is crucial for effector T cell proliferation and differentiation.^17,31,117^ In addition, mTORC1 sustains glycolysis that supports Th17 cell (but antagonizes Treg cell) differentiation by upregulating HIF-1α expression,^118,119^ which requires upstream PDK1 but not PI3K signaling.^25^

By regulating the activation of AGC kinases, mTORC2 activity is also linked to metabolic reprogramming, although its function seems to be less important for early metabolic programming (i.e., that occurs during initial quiescence exit) that promotes T cell activation.^31^ As such, mTORC2 can be considered as an amplifier of mTORC1-related metabolic reprogramming. As an example, mTORC2 activity supports the optimal induction of glycolysis, by inducing Akt activity that promotes surface expression of GLUT1, which regulates Th1 and Tfh cell differentiation.^29,34,35,70^ Interestingly, mTORC2 activity also promotes Th2 cell differentiation, which also requires glycolysis,^31^ via the PKC-dependent induction of GATA3 expression.^29^ mTORC2 signaling can also regulate the Akt-dependent inactivation of GSK-3 at ER–mitochondria contacts to enable pyruvate oxidation in mitochondria.^120^ The metabolic programs shaped by extracellular minerals, such as sodium, are also likely to be regulated by mTORC2. For example, SGK1 is activated by mTORC2, and SGK1 expression is induced under conditions of elevated salinity and promotes the generation of pathogenic Th17 cells by maintaining expression of IL-23 receptor.^121,122^ SGK1 also positively regulates Th2 cell differentiation while suppressing Th1 cell development.^123^ The molecular mechanisms for selectivity of mTORC2 target activation in T cells await discovery.

mTOR signaling also plays pleiotropic roles in regulating mitochondrial metabolism. Mitochondrial metabolism in naïve T cells is catabolic, which supports cellular homeostasis.^2^ Under these conditions, mTOR signaling is actively retained at lower levels, which establishes the quiescence of these cells.^68^ Naïve T cells also have low mitochondrial content that increases as the cells differentiate into effector T cells.^66,124^ mTORC1 activation promotes mitochondrial biogenesis via the induction of several mitochondrial proteins, including mitochondrial ribosomal subunits.^66^ These effects may also be mediated by the mTORC1–4E-BP1-dependent upregulation of Tfam (transcription factor A, mitochondrial), which promotes expression of mitochondrial DNA-derived genes (e.g., selective components of the electron transport chain).^125^ Mitochondrial function is further increased as cells transition from effector to memory T cells; this enhanced mitochondrial function is mediated, in part, by mitochondrial fusion driven by CD28 co-stimulation.^126–128^ This event is also likely associated with decreased mTOR signaling,^76–79^ but the precise mechanisms that govern downregulation of mTOR signaling for the effector-to-memory T cell transition remain unknown.

The mTORC1-dependent upregulation of mitochondrial metabolism promotes efficient OXPHOS, as well as the generation of different epigenetic-regulating metabolites (e.g., α-KG, 2-hydroxyglutarate and acetyl-CoA) to regulate T cell functional programming.^17,66,129–135^ Another consequence of increased mitochondrial biogenesis is augmented serine- and folate-dependent one-carbon metabolism, which regulates the generation of the methyl-donor S-adenosylmethionine (SAM), as well as glutathione and nucleotides important for T cell activation.^124,136–140^ In T cells, the upregulation of one-carbon metabolism-associated enzymes requires mTORC1 activation,^66^ which may be mediated by the mTORC1-dependent activation of ATF4.^141^ In summary, mTOR activity coordinates transcriptional and post-transcriptional programming to mediate anabolic metabolism for control of T cell responses.

### Lipid and cholesterol synthesis

Lipids and cholesterol are integral components of cellular membranes and are important regulators of T cell responses. De novo lipid and cholesterol synthesis promotes activation-induced proliferation and differentiation of T cells. This metabolic shift is partially controlled by mTORC1 downstream of TCR activation, as Raptor-deficient naïve CD4^+^ T cells have lower expression of lipid synthesis-related genes (e.g., *Hmgcr* (encodes HMG-CoA reductase, HMGCR; rate-limiting enzyme for mevalonate synthesis), *Fasn* and *Scd*), associated with reduced proliferation and cell growth.^31^ These effects also extend to Raptor-deficient Treg cells.^72^ Unlike mTORC1, the role of mTORC2 in lipid metabolism is largely unknown in immune cells, but some studies suggest a role in the de novo synthesis of sphingolipids by regulating the activity of ceramide synthase.^142^ Interestingly, ceramide synthesis is involved in promoting efficient TCR signaling, activation and effector responses in human T cells,^143–145^ which appears to be linked with mitochondrial respiration through unknown mechanisms.^135^

Fatty acid synthesis is initiated by the cytosolic enzyme acetyl-CoA carboxylase-1 (ACC1), which promotes the conversion of mitochondria-derived acetyl-CoA into malonyl-CoA, the precursor for fatty acid synthesis. ACC1 deficiency in T cells inhibits Th17 and induces Treg cell differentiation in vitro, which is recapitulated using the ACC1 inhibitor soraphen A.^146^ Notably, both pharmacologic or genetic inhibition of ACC1 not only blocks fatty acid synthesis, but also impedes metabolic flux through glycolysis and the tricarboxylic acid cycle,^146^ possibly due to accumulation of metabolic intermediates (e.g., citrate or others) that enforce feedback inhibition of these pathways. Deficiency for ACC1 also impairs Th1 and Th2 cell development.^146^ On the other hand, ACC1 deletion in T cells does not affect CD8^+^ T cell function, even though it enhances cell death and reduces effector T cell expansion in response to bacterial infection.^147^ These findings suggest that fatty acid synthesis is important for CD8^+^ T cell persistence. The mitochondrial isoform ACC2 is involved in balancing lipid metabolism in the mitochondria through inhibition of carnitine palmitoyl-transferase a (CPT1a)-dependent fatty acid β-oxidation (FAO),^148^ but this effect is not essential for CD8^+^ T cell function.^149^

In T cells, mTORC1 induces de novo lipid and cholesterol synthesis by increasing the respective expression of sterol regulatory element-binding protein-1 (SREBP1; isoforms SREBP1-a and SREBP1-c) and SREBP2, which may be connected to the cholesterol-dependent activation of mTORC1.^31,108^ mTORC1 also phosphorylates lipin-1, which promotes the nuclear localization of lipin-1 and allows for the induction of SREBP expression and function.^150^ The activity of SREBPs is also directly controlled by the cholesterol-sensing protein SREBP cleavage-activating protein (SCAP). Specifically, when cholesterol concentrations are high, SCAP sequesters SREBP in the ER. Upon cholesterol depletion, the SCAP–SREBP complex moves to the *trans*-Golgi, where SREBPs are cleaved by the proteases S1P and S2P to allow for SREBP translocation from the Golgi complex to the nucleus, where they regulate the expression of lipogenesis-inducing genes and transporters.^151,152^ Studies have shown important roles for SREBP function in T cells. Deletion of SCAP in T cells does not affect homeostatic proliferation, but impairs activation-induced cell growth, proliferation and differentiation, which promotes clearance of viral infections in vivo.^153^ The reduction of cell growth and proliferation is restored upon addition of exogenous cholesterol, which supports membrane biogenesis.^153^ Through c-Myc, HIF-1α and AMPK-independent mechanisms, SCAP deficiency also inhibits metabolic reprogramming towards glycolysis, glutaminolysis and mitochondrial function,^153^ which may further impede lipid synthesis by limiting the concentrations of upstream, pro-lipogenic metabolites (e.g., acetyl-CoA). Thus, SREBPs are multifactorial regulators of metabolic programming in activated T cells.

How do activated T cells maintain SREBP function when the accumulation of intracellular cholesterol may impede SREBP activity? First, the expression of SULT2B1, which is an oxysterol-metabolizing enzyme, is upregulated upon T cell activation.^154^ Consequently, oxysterols that may be metabolized from cholesterol are unable to activate the liver X receptor (LXR) transcription factors, which promote cholesterol efflux by increasing expression of the ABCG1 transporter.^154,155^ LXRβ-deficient T cells have increased cellular proliferation, whereas deletion of ABCG1, but not the related protein ABCA1, reduces T cell proliferation to the same extent as LXR activation,^154^ demonstrating functional antagonism of SREBP and LXR in T cell proliferation. These effects are probably linked to ABCG1-dependent suppression of mTOR activity.^156^ Second, cholesterol derivatives can modulate the strength or duration of TCR signaling, with cholesterol esters and cholesterol sulfate decreasing TCR signal strength.^157,158^ That reducing cholesterol esters does not alter glycolysis or mitochondrial oxygen consumption, despite the increase of TCR signaling,^158^ may suggest inhibitory roles for cholesterol derivatives on mTOR activation, which remains to be addressed.

Fatty acids also play signaling roles to sustain pro-lipogenic programming apart from SREBP. For instance, fatty acids activate peroxisome proliferator-activated receptors (PPARs), which are nuclear hormone receptors that are important for lipid metabolism, as well as glucose homeostasis in cells. CD28 co-stimulation augments mTORC1 activation, as well as fatty acid uptake, to increase PPARγ expression and activity, respectively, in T cells.^159^ Further, PPARγ-deficient CD4^+^ T cells have reduced proliferation, survival and Th17 cell differentiation, which is associated with attenuated autoimmune and graft-vs.-host disease responses in mice.^159,160^ These features are also associated with reduced metabolic reprogramming upon TCR stimulation, and the defects in activation are restored by exogenous fatty acids.^159^

Fatty acids differ by length, with those of total carbon atom numbers from 1 to 6 usually categorized as short-chain fatty acids (SCFAs), whereas those of 7–12 carbon atoms are defined as medium-chain fatty acids; long-chain fatty acids (LCFAs) have more than 12 carbons.^161^ The microbiota-derived SCFA butyrate promotes cellular metabolism, by enhancing the memory differentiation potential of activated CD8^+^ T cells; butyrate also promotes optimal recall responses upon antigen re-stimulation,^162^ an effect that has also been observed with the glycolysis-inducing SFCA acetate.^163^ Intestine-derived SCFAs also support Treg cell generation in non-lymphoid tissues.^164–166^ Dietary LCFAs alter the composition of the gut microbiome.^167^ Further, LCFAs enhance Th1 and Th17 cell differentiation whereas SCFAs promote Treg cell formation,^168^ demonstrating unique phenotypes driven by different fatty acids.

Recent work has indicated that de novo-synthesized lipids can also drive catabolic functions of certain T cell subsets. For example, memory CD8^+^ T cells display less LCFA uptake than effector CD8^+^ T cells.^169^ Instead, upon IL-7 stimulation, memory CD8^+^ T cells can increase expression of aquaporin 9 for glycerol import to promote synthesis of triacylglycerides (TAGs) and sustain ATP production via FAO.^170^ Thus, TAG synthesis is an important process for modulation of memory T cell survival and self-renewal, which could possibly be exploited for therapeutic manipulation of these processes.

### Autophagy

Autophagy is an evolutionarily-conserved pathway that promotes degradation of cytoplasmic components. This process is initiated through the formation of a double-membraned, intracellular organelle called an autophagosome, which, through a series of biochemical reactions, fuses with the lysosome for degradation and recycling of internal proteins and organelles.^63^ When nutrient concentrations are abundant, mTORC1 impairs autophagic flux^171^ by directly inhibiting the activity of the ULK complex (ULK1, Atg13 and FIP200), which is required for autophagy.^172,173^ mTORC1 can also indirectly affect autophagy at the level of lysosome biogenesis. Specifically, mTORC1 suppresses lysosome biogenesis via inhibition of transcription factor EB (TFEB) family transcription factors, which induce expression of numerous lysosome- and autophagy-specific genes.^174^ On the other hand, under nutrient restriction, mTORC1 activity is suppressed, leading to the activation of the ULK complex and TFEB transcription factors, thus permitting the induction of autophagy.

Autophagy is essential for T cell homeostasis, function and differentiation. For example, deletion of Atg7 in mature T cells reduces cell survival. In addition, compared to naïve T cells, autophagic flux is higher in antigen-specific CD8^+^ T cells.^175^ During acute infection of LCMV, autophagy is downregulated in CD8^+^ T cells undergoing proliferative expansion, whereas autophagy is upregulated before the contraction phase of the response. Deletion of the autophagy proteins Atg5 or Atg7 in effector CD8^+^ T cells is associated with reduced cell survival and memory CD8^+^ T cell generation.^175,176^ Autophagy also supports T cell function in anti-tumor immunity.^177^ In addition, autophagy is upregulated in Treg cells compared to naïve CD4^+^ T cells, and it promotes Treg cell responses for suppression of autoimmunity, anti-tumor immunity and other inflammatory disorders.^26,178^ These effects are attributed, in part, to reduced Treg cell survival in the absence of autophagy. Of note, we have recently found that suppression of autophagy in Treg cells is associated with an increase of mTORC1 activity and glycolysis, partially due to defects in downregulating expression of PI3K-related signaling components.^26^ Heightened glycolysis has also been observed in autophagy-deficient CD8^+^ T cells.^177^ Thus, autophagy supports many aspects of T cell fitness and function.

### Emerging perspectives

Several proteins, such as the Tsc complex, Rheb, Rag, Raptor and Rictor, are conserved signaling nodes that influence mTOR activation in T cells.^29–31,68,76,112,116^ What remains to be determined is whether unique protein sensors of TCR-independent signaling networks (e.g., ‘signal 3′ cytokines) or other nutrients aside from amino acids exist, which may directly promote mTORC1 activation in T cells. In addition, besides the Tsc complex,^68,76^ the T cell-specific proteins that directly antagonize mTOR activity in response to immunological signals and nutrients are largely unknown. These may also include sensors of metabolites, such as those recently identified for SAM,^179^ which may suppress (or activate) mTORC1 to impart specificity of T cell responses. Another aspect that has remained unclear is how mTORC1-dependent signals are transmitted to functional T cell responses. For example, how can complete abrogation of mTORC1 signaling via Raptor deletion inhibit mitochondrial biogenesis, while incomplete suppression of mTORC1 signaling via Rheb deletion increase mitochondrial synthesis?^66,76^ One possible explanation for how ‘graded’ reductions in mTORC1 signaling alter metabolic programs, and ultimately cellular fate decisions, may lie in the discrete regulation of downstream targets. Indeed, we have shown that the phosphorylation of 4E-BP1 is more resistant to Rheb deletion than S6K, which is associated with milder defects in T cell activation than Raptor deficiency;^31^ these observations are consistent with those showing that the 4E-BP1 axis more selectively promotes the growth and proliferation of lymphocytes than the S6K pathway.^180^ Similarly, how mTORC2 targets affect T cell function in different physiological contexts will be interesting to define, as studies have implicated unique roles of Akt, PKC and SGK1 in T cell responses.^29,121–123^ Finally, the identification of novel mTOR targets in T cells may be achievable using mass spectrometry-based approaches.^66,181,182^ Such discoveries may uncover new therapeutic targets wherein selective aspects of mTOR signaling may be inhibited without detriment to others, such as dictating suppression of pro-inflammatory T cell responses without suppressing Treg cell function that is important for establishing immunological tolerance.^29–31,72,73,76,112,116^

## LKB1–AMPK signaling

**Fig. 3:**
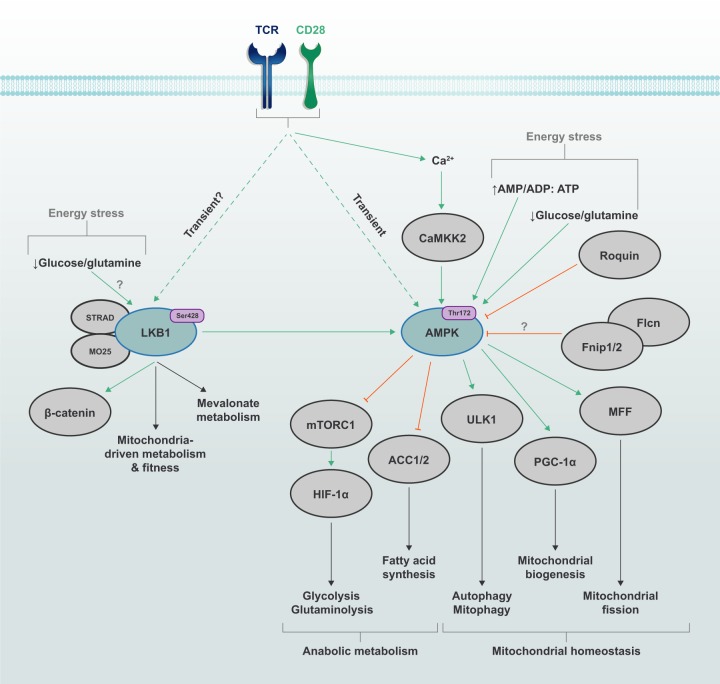
Metabolic programming in T cells through LKB1 and AMPK signaling. The energy stress pathway kinases LKB1 and AMPK are activated by TCR and CD28 co-stimulatory signals, with AMPK activity being mediated, in part, by the Ca^2+^–CAMMK2 pathway. Energy stress, such as deprivation of glucose or glutamine or an imbalance of AMP/ADP-to-ATP ratio, can also promote LKB1–AMPK signaling. Upstream nutrient-sensing proteins, such as the Fnip–Flcn complex and Roquin, can restrain AMPK function, although the contribution of Fnip and Flcn to AMPK signaling in T cells is still unknown. The activation of LKB1 is associated with changes in mitochondrial metabolism and fitness, as well as increased mevalonate metabolism under certain contexts. By regulating the activity of several downstream targets, AMPK signaling can impede metabolic programming toward glycolysis, glutaminolysis and fatty acid synthesis, while promoting catabolic processes, such as mitophagy and autophagy. AMPK also supports mitochondrial fitness by driving mitochondrial biogenesis and mitochondrial dynamics by promoting mitochondrial fission.

The LKB1–AMPK signaling pathway plays a central role in regulating cellular metabolism, proliferation and survival in response to altered nutrient and energy demands. LKB1–AMPK signaling promotes catabolic pathways that produce ATP, and enables metabolic plasticity in T cells in response to energy stress. By regulating metabolic reprogramming, LKB1 and AMPK contribute to T cell differentiation and function. In this section, we describe how LKB1 and AMPK activities are regulated, their effects on metabolism and roles in T cell-mediated immunity (Fig. 3).

### Regulation of LKB1 and AMPK activities

LKB1 is a serine/threonine kinase that functions as a tumor suppressor and is involved in the regulation of cellular metabolism and proliferation. LKB1 has many downstream targets, including the most well-defined target AMPK and AMPK-related kinases, such as BRSK, NUAK and MARK.^183–185^ The upstream regulation of LKB1 is mediated by cellular localization and post-translational modifications. LKB1 forms a complex with STE20-related adapter (STRAD) and MO25, which promotes the cytoplasmic localization and kinase activity of LKB1, respectively.^185,186^ Further, Akt can inhibit LKB1 by promoting LKB1 nuclear retention.^187^ LKB1 undergoes many post-translational modifications, including phosphorylation, mevalonate pathway-related protein farnesylation, ubiquitination, SUMOylation and acetylation; however, how many of these modifications regulate LKB1 activity and the modifying enzymes required are unknown.^185^ Further, the contribution of many upstream regulators to LKB1 signaling in T cells remains underexplored.

AMPK is a conserved serine/threonine kinase that is comprised of α-, β- and γ-subunits. AMPK signaling is regulated by intracellular energy levels by sensing of the AMP/ADP:ATP ratio.^188^ AMP competes with ATP for binding to the cystathionine β-synthase 1 (CBS1) repeat in the γ-subunit of AMPK, which allosterically activates AMPK. Moreover, AMP and ADP binding to the CBS3 repeat limits access of phosphatases to Thr172 on the AMPK catalytic α-subunit, the critical phosphorylation site that promotes AMPK kinase activity.^188,189^ Altered nutrient availability can also regulate AMPK. Glucose- and glutamine-deficient conditions induce acute AMPK activation in T cells without altering intracellular ATP levels.^190^ Glucose starvation also promotes AMPK activation independently of AMP/ADP by reducing intracellular concentrations of fructose-1,6-bisphosphate (FBP).^191^ Low FBP levels allow the FBP enzyme aldolase to promote association of LKB1–Axin with the lysosomal/endosomal SLC38A9-v-ATPase-Ragulator network for the subsequent activation of AMPK;^95,191^ whether FBP deprivation leads to AMPK activation in T cells is unknown. Ultimately, the intricate regulation by metabolites allows for a ‘graded’ response of AMPK activity under varying energy stress conditions.

At least three upstream kinases mediate AMPK Thr172 phosphorylation: LKB1, calcium calmodulin kinase kinase 2 (CaMKK2) and TGF-β-activating kinase 1 (TAK1), with roles for LKB1 and CaMKK2 being most understood. As previously mentioned, LKB1 serves as the major kinase acting on Thr172.^192–194^ Further, through regulation by CaMKK2,^195–197^ AMPK may play a role in Ca^2+^-mediated signaling. In T cells, both TCR and Ca^2+^ signaling, along with CD28 co-stimulation, coordinate to rapidly and transiently activate AMPK through CaMKK2.^198–200^ AMPK activity is also regulated through protein-protein interactions. Fnip1 and Fnip2 form a complex with AMPK, and a loss-of-function mutation in Fnip1 elevates AMPK activity in B cells, establishing Fnip1 as a negative regulator of AMPK.^201^ The consequence of impaired Fnip1 function is a profound loss in B cells due to dysregulated AMPK and mTOR signaling.^201,202^ Additionally, Fnip1 promotes invariant natural killer T cell development in the thymus, potentially through maintaining intracellular ATP levels.^203^ The Fnip-binding protein Flcn also interacts with AMPK and may serve as a negative regulator of AMPK signaling, and there is some evidence of reciprocal regulation of Fnip1 and Flcn by AMPK.^204,205^ Beyond the Fnip–Flcn complex, Roquin directly binds the AMPKα1 subunit and limits AMPK signaling, likely through the sequestration of AMPK to stress granules,^206^ and Roquin-mediated regulation of AMPK is required for Tfh cell responses.^206^ The contribution of the Flcn–Fnip1–AMPK signaling axis in T cell biology remains poorly defined.

### Catabolic programming mediated by LKB1–AMPK

Consistent with its induction by low nutrient abundance, AMPK activity is often associated with the programming of catabolic metabolism to generate ATP. AMPK can directly phosphorylate Raptor at two serine sites (Ser722 and Ser792 of human Raptor), which promotes 14-3-3 binding to Raptor and disrupts mTORC1 signaling.^207^ Further, the LKB1–AMPK kinase cascade phosphorylates Tsc2 at a site that promotes its GAP activity and thus inhibits mTORC1 signaling.^208^ In contrast to the inhibitory role of mTORC1 described earlier, AMPK activates autophagy via the phosphorylation of ULK1.^209^ ULK1 can contribute to autophagy-mediated mitochondrial homeostasis to promote T cell survival.^210^ Further, independent of mTOR regulation, AMPK also mediates cell cycle arrest through phosphorylation of p53 at Ser15 when glucose is limiting.^211^ Thus, LKB1–AMPK signaling promotes cell survival by limiting anabolic metabolism and cellular growth during energy stress.

AMPK can also inhibit the synthesis of fatty acids and cholesterol by regulating the enzymes ACC1, ACC2 and HMGCR.^212^ AMPK phosphorylation of ACC1 Ser79 and ACC2 Ser212 inhibits the conversion of acetyl-CoA to malonyl-CoA, the precursor for fatty acid synthesis.^212,213^ Mice with mutated Ser79 and Ser212 sites on ACC1 and ACC2, respectively, have elevated lipogenesis and reduced FAO,^213^ demonstrating that AMPK-dependent inhibition of ACC1 and ACC2 promotes FAO. Further, disruption of the LKB1–AMPK pathway by the metabolite ribulose-5-phosphate, generated through the pentose phosphate pathway, promotes lipogenesis.^214^ However, LKB1 can also promote mevalonate metabolism to support Treg cell homeostasis in an AMPK-independent manner.^215^ Thus, LKB1–AMPK signaling primarily acts to suppress lipid synthesis pathways, but AMPK-independent LKB1 signaling may promote de novo lipogenesis in selective contexts.

As noted above, catabolic programs driven by mitochondria support quiescent T cell homeostasis, which can be fueled, in part, by FAO.^2^ Some subsets of memory CD8^+^ T cells express high levels of CPT1a, which is a mitochondrial transporter of LCFAs.^127^ AMPK induces FAO in memory CD8^+^ T cells, and deficiency for tumor necrosis factor (TNF)-associated factor-6 (TRAF6; signals downstream of TNF receptor superfamily and other immunological receptors) severely impairs AMPK signaling and FAO in CD8^+^ T cells after IL-2 withdrawal, which induces unchecked metabolic stress and corresponds to impaired memory CD8^+^ T cell development.^216^ Importantly, in vivo treatment of TRAF6-deficient mice with the AMPK agonist metformin partially restores memory CD8^+^ T cell generation.^216^ Further, AMPKα1 is essential for memory CD8^+^ T cell recall responses.^217^ Thus, AMPK signaling promotes lipid metabolism for the generation of a functional memory CD8^+^ T cell population.

More recent studies using genetic mouse models have questioned the role of FAO in T cell subsets. In contrast to shRNA knockdown or pharmacological inhibition of CPT1a by etomoxir, mice with T cell-specific deletion of *Cpt1a* do not exhibit defective generation of memory CD8^+^ T cells or Treg cells.^218^ Further, mice with Treg cell-specific CPT1a deficiency show normal immune homeostasis, suggesting that CPT1a-dependent FAO is dispensable for Treg cell function for establishment of immune tolerance in vivo.^218,219^ These discrepancies may be, in part, attributable to off-target effects of high-dose etomoxir, such as depletion of coenzyme A levels that are essential for driving induction of fatty acid synthesis among other functions.^220^

Memory T cell responses are also important for anti-tumor immunity. The induction of HIF-1α activity via deletion of the von Hippel-Lindau protein promotes glycolysis, which induces effector memory T cell generation and function.^221^ Studies in tumor cells have demonstrated that the disruption of LKB1 or AMPK signaling promotes aerobic glycolysis, in part, through HIF-1α, which results in increased transcription of glycolytic enzymes.^222–225^ LKB1–AMPK-dependent regulation of HIF-1α may also partly depend on suppression of mTORC1.^226^ LKB1–AMPK signaling may indirectly orchestrate the differentiation of Th17 and Treg cell lineages through HIF-1α- or ACC1-mediated changes in glycolysis and mitochondrial oxidative metabolism.^118,119,146^ Additionally, recent work demonstrated that LKB1 promotes stable Foxp3 expression,^215,227^ as well as Th2-like Treg cell development independently of AMPK^215,228^ and mTORC1–HIF-1α signaling but dependent on β-catenin signaling.^228^ LKB1 signaling is required for mitochondrial function and mitochondria-dependent metabolic programs upon TCR-mediated Treg cell activation,^228^ including FAO or purine and pyrimidine metabolism.^229^ These findings highlight that LKB1 and AMPK orchestrate metabolic reprogramming to regulate T cell differentiation and Treg cell function.

### AMPK signaling and adaptation to metabolic stress in T cells

Adaptive immune responses are metabolically demanding and require adaptation to nutrient and metabolic alterations to support their survival and proliferative expansion at sites of activation and infection. As noted above, mTORC1 signaling, combined with mTORC2 activity, coordinates many of the initiating events that are necessary to meet these metabolic demands.^31,66^ AMPK also enables T cell metabolic adaptation, which can occur independently of TCR signaling. For instance, T cells in glucose-depleted conditions have impaired cellular proliferation, survival and function,^60,230^ and the absence of AMPKα1 further enhances cell death.^190^ AMPK promotes T cell survival by supporting glutaminolysis and mitochondrial OXPHOS to maintain intracellular ATP levels in the absence of glucose by promoting the expression of genes involved in glutamine uptake and metabolism.^190^ Further, AMPK regulates mitochondrial homeostasis through PGC-1α-mediated mitochondrial biogenesis and by phosphorylating mitochondrial fission factor to initiate mitochondrial fission,^231,232^ which may allow for sustained glycolysis and anti-tumor function of T cells.^126,233^ In addition, AMPK mediates recycling of damaged mitochondria through ULK1,^234^ a process that can be induced by elevated production of mitochondria-derived reactive oxygen species.^235^ AMPKα1 deficiency consequently impairs primary effector CD8^+^ T cell responses to viral and bacterial infections in vivo, or the expansion of CD4^+^ Th1 and Th17 cells in lymphopenic environments.^190^ Thus, AMPK controls metabolic reprogramming during nutrient starvation and mitochondrial homeostasis to promote effector T cell responses.

### Emerging perspectives

Understanding the regulation of the LKB1–AMPK signaling axis is an important area of immunological research for several reasons. T cells must adapt to inflammatory and nutrient-depleted conditions, such those that occur in the tumor microenvironment;^60,236^ therefore, pathways such as AMPK that mediate metabolic flexibility are likely to have critical implications in adoptive T cell therapy. While LKB1 phosphorylates many kinases, AMPK has been the primary target examined in most studies. However, there is emerging evidence that LKB1 has AMPK-independent roles in T cell biology, including Treg cell-dependent suppression of autoimmunity and T cell-dependent inhibition of intestinal polyp formation.^215,228,237^ Thus, the contribution of LKB1 downstream targets in T cell biology and metabolism is an exciting area that requires more exploration.

An emerging field in immunology is the regulation of T cell biology by nutrient and metabolite signaling. The LKB1–AMPK axis serves as a critical signaling nexus to integrate metabolic cues for T cell function and fate. For instance, several targets of LKB1 and AMPK are implicated in epigenetic regulation of chromatin accessibility, such as by the protein deacetylase sirtuin 1 (Sirt1). AMPK enhances Sirt1 activity by increasing intracellular NAD^+^ levels,^238^ which may lead to altered chromatin structure through histone deacetylation, as well as protein activity through post-translational modifications. In T cells, AMPK may promote memory CD8^+^ T cell development, in part, through Sirt1, which can regulate human memory CD8^+^ T cell metabolism and cytotoxic function through FoxO1 that is implicated in T cell differentiation and memory CD8^+^ T cell formation.^238–241^ However, the role of Sirt1 for epigenetic reprogramming in T cells remains to be explored. Thus, the contribution of downstream mediators of LKB1–AMPK signaling to impart metabolic regulation of T cell fate is an exciting area for future studies.

## Immunometabolic signaling networks

The major signaling pathways described above are not functionally exclusive; rather, they are intertwined and reciprocal, together culminating in the proper metabolic regulation to meet context-specific needs for cellular function. As such, many of these regulatory inputs converge at common nodes, which include both individual molecules and entire cellular processes as discussed below.

### Integration of signaling pathways to regulate key cellular and metabolic nodes

**Fig. 4:**
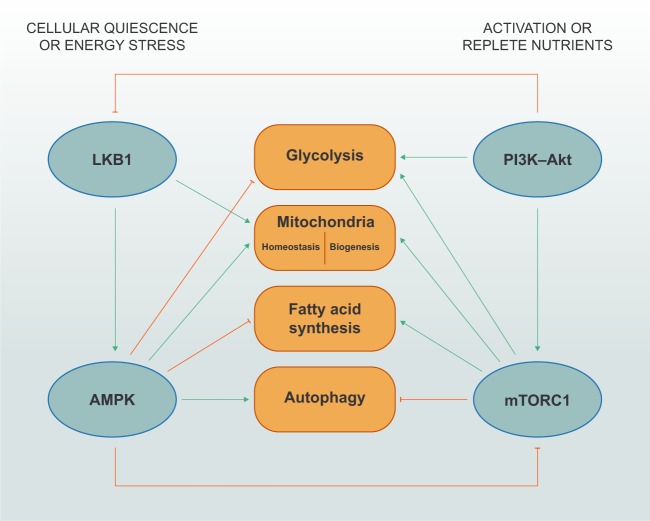
Cross-regulation of immunometabolic signaling pathways. Under nutrient-deprived conditions, LKB1–AMPK signaling inhibits anabolism-associated programs, such as glycolysis and fatty acid synthesis, while promoting mitochondrial homeostasis and autophagy; however, these mechanisms require additional investigation in primary T cells. AMPK directly inhibits mTORC1 through phosphorylation of its obligate adapter protein Raptor (not depicted). During activation and/or replete nutrient conditions, PI3K–Akt and mTORC1 signaling promotes glycolysis, mitochondrial biogenesis and fatty acid synthesis, while inhibiting autophagy. Akt can reportedly phosphorylate LKB1 to suppress its functional localization, but this regulation is not yet reported to occur in T cells.

In general, elevated activity of PI3K–AGC kinases and mTOR, together with decreased LKB1–AMPK activity, is associated with cell growth, proliferation and effector function, which is associated with changes in metabolic programming as summarized in Fig. 4. The crosstalk between these signaling pathways can occur through the direct reciprocal antagonism, such as between mTORC1 and AMPK^190,207,242^ or Akt and LKB1,^187^ or via indirect inhibition of downstream signaling effectors. For example, the ULK1 complex, which promotes autophagy, is inhibited and activated by mTORC1 and AMPK signaling, respectively.^172,173,234,235^ Further, autophagy may act as an ‘off-switch’ for mTORC1 activation and glycolysis under nutrient-deficient conditions.^26^ This inhibition likely favors cell survival and maintains cellular quiescence at the expense of cell growth and proliferation.^243^ Understanding the mechanisms for the interplay between autophagy and mTORC1 may be therapeutically relevant for immune-related diseases, such as autoimmune disorders and cancer.

The integration of signaling by upstream complexes is ultimately linked to regulation of downstream metabolic programming. For instance, LKB1–AMPK signaling inhibits fatty acid synthesis and mevalonate metabolism in certain contexts through the direct regulation of ACC1 or ACC2 and HMGCR,^212,213^ while mTORC1 signaling promotes the function of these molecules in T cells by upregulating gene expression downstream of the SREBP transcription factors.^31,153^ However, it is important to note that although AMPK can promote FAO in T cells,^216^ it is unclear whether AMPK negatively regulates fatty acid synthesis in T cells. AMPK signaling can also oppose glycolysis through negative regulation of mTORC1–HIF-1α signaling.^222–226^ However, when glucose is limiting, AMPK signaling can also promote glutamine metabolism and flux via the mitochondrial TCA cycle in T cells.^190^ Also, LKB1 and AMPK signaling can influence mitochondrial fitness and biogenesis in a potentially mTORC1-independent manner.^228,229,231,232^ Thus, the crosstalk between PI3K–Akt, mTOR, LKB1 and AMPK signaling (along with other immunometabolic signaling networks, such as those mediated by Regnase-1^244^), likely promotes metabolic adaptations that allow cells to meet energetic and biosynthetic demands to function in unique microenvironments.

The signaling pathways that dictate metabolic programs in immune cells are also reciprocally influenced by metabolites and nutrients. This ‘bidirectional metabolic signaling’ provides another layer of regulation on kinase-dependent signaling and gene transcription to control immune cell function in different contexts.^2^ As described above, mTORC1 signaling is dynamically regulated by nutrients and metabolites, with amino acids promoting mTORC1 activation through modulation of upstream proteins such as the Rag complex.^63,112,116^ The subcellular localization of nutrients can also influence signaling and metabolic programming, as shown by recent observations that TCR stimulation inhibits pyruvate translocation into the mitochondria, leading to the promotion of aerobic glycolysis.^32^ Conversely, diminished nutrient conditions can regulate signaling pathways to control metabolism. For example, low cellular glucose and glutamine can activate AMPK.^190^ In addition, elevated AMP or ADP concentrations promote AMPK activation, which limits anabolism and promotes mitochondrial homeostasis to replenish ATP stores.^188^ Finally, several key metabolites derived from mitochondria-associated metabolism, including α-KG, 2-hydroxyglutarate and acetyl-CoA, serve as co-factors for epigenome-modifying enzymes to influence transcriptional activity.^2^ Thus, the metabolites generated as a consequence of kinase-dependent metabolic signaling also have signaling roles to impart effects on cellular function.

## Concluding remarks

The role of immune signaling networks in immunometabolism is an exciting area of research, with broad implications for therapy and human health. In this review, we highlighted the critical roles for PI3K–AGC, mTOR and LKB1–AMPK signaling in T cell function and fate. While the field has made much progress in understanding how these signaling molecules are regulated and how they reprogram T cell metabolism, many outstanding questions still remain, including: (1) Do altered receptor signal strength and duration modulate T cell responses through different downstream targets? (2) Do specific upstream regulators and downstream targets of AGC kinases, mTOR and LKB1 transmit nutrient and immunological signals to define T cell subset-specific metabolic programs? (3) How do upstream signaling networks (e.g., LKB1–AMPK) integrate metabolite sensing with T cell metabolic and epigenetic programming? In addition, the roles of other signaling networks, including the conserved Hippo family kinases and mitogen activated-protein kinase pathways, in immunometabolism are only beginning to be understood.^245,246^ Technical advances and systems immunology approaches should also accelerate our understanding of how these signaling networks crosstalk with metabolic programs to control T cell outcomes. Finally, determining how relatively well-known upstream immune signals (e.g., TCR, cytokine receptors) impart different functional programs on metabolism may provide new therapeutic targets for improving adoptive T cell therapy, such as anti-tumor CAR-T cell therapy.
